# Impact of the Food-Related Stress Conditions on the Expression of Enterotoxin Genes among *Staphylococcus aureus*

**DOI:** 10.3390/pathogens12070954

**Published:** 2023-07-19

**Authors:** Joanna Gajewska, Arkadiusz Józef Zakrzewski, Wioleta Chajęcka-Wierzchowska, Anna Zadernowska

**Affiliations:** Department of Industrial and Food Microbiology, Faculty of Food Science, University of Warmia and Mazury in Olsztyn, 10-726 Olsztyn, Poland; arkadiusz.zakrzewski@uwm.edu.pl (A.J.Z.); wioleta.chajecka@uwm.edu.pl (W.C.-W.); anna.zadernowska@uwm.edu.pl (A.Z.)

**Keywords:** *S. aureus*, gene expression, enterotoxin gene

## Abstract

*Staphylococcus aureus* is one of the most important foodborne pathogens. *S. aureus* has the capability to produce a variety of toxins, including staphylococcal enterotoxins (SEs). The aim of this study was to evaluate the survival rate of *S. aureus* cells and analyze enterotoxins gene expression after exposure to osmotic stress and acidic/alkaline stress. To determine survival rates, the traditional plate counting method and flow cytometry were used. The expression levels of the enterotoxin genes were performed by quantitative reverse transcription PCR (RT-qPCR). Expression changes differed depending on the stressors chosen. The obtained results in this study showed the effect of critical food-related stress conditions on SE gene expression in *S. aureus*. The study showed different expression levels of the tested enterotoxins genes depending on the stress. The most tested enterotoxin genes (*seg*, *sei*, and *selo*) after exposure to pH = 4.5 stress have similar expression as in the optimal condition. After alkaline treatment (pH = 9.6), a similar expression gene value as for the optimal condition was observed. The analysis of gene expression in response to stress caused by NaCl, showed that the expression of *selp* decreased, whereas *selu*, *selm*, and *selo* genes increased. A significantly decreased expression of the *sea* gene was observed.

## 1. Introduction

Staphylococcal food poisoning (SFP) belongs to one of the most frequently occurring food poisonings globally, caused by eating food contaminated with staphylococci enterotoxin (SEs) secreted by *Staphylococcus aureus* [1]. In the European Union, the number of SFP cases is decreasing, with 43 outbreaks, 402 cases, and 32 hospitalizations reported in 2020, whereas in 2021 there were only 13 reported outbreaks caused by staphylococcal toxins [2,3].

The literature data report that foods of animal origin, such as milk and dairy products, are the cause of SFP. It is worth emphasizing that staphylococcal food poisoning is one of the most common foodborne illnesses worldwide [4]. Nowadays, over 20 different enterotoxins have been characterized [5]. These enterotoxins were subdivided into two groups. The first of these includes SEA-SEE, called classical enterotoxins (SEs) with demonstrated emetics activity, and the second one is staphylococcal enterotoxin-like proteins (SEls), which are potential emetics in a primate model or have yet to be tested [5,6,7]. Enterotoxins encoding genes can be located on various mobile genetic elements, such as transposons, plasmids, prophages, pathogenicity islands (SaPIs), and highly variable genomic regions (vSa). The role of SE genes as virulence determinants in *S. aureus* is thought to be regulated by the *agr* (accessory gene regulator) gene and *sar* (staphylococcal accessory regulator) gene, alternative sigma factor B (σ^B^), and repressor of toxins (Rot). [8,9]. Enterotoxins have the capability to stimulate large populations of T cells, leading to unregulated immune system activation [10]. The most common symptoms of SFP include acute gastroenteritis, diarrhea, and vomiting, which occur within 2 to 6 h after consumption of food containing SEs [1,11]. The SFP is a mild, self-limiting disease. Symptoms usually resolve within 12 to 24 h [10]. The most frequently identified enterotoxin genes in SPF have been documented to be *sea*, *seb*, and *sed*, although this pattern appears to be evolving over time. A study carried out by Omoe et al. (2013) [12] demonstrated that the SEK, SEL, SEM, SEN, SEO, SEP, and SEQ enterotoxins, following oral administration, also induce vomiting in monkeys (*Macaca fascicularis*). The authors showed that a dose (of all enterotoxins) of 100 μg/kg caused a vomiting response in the tested monkeys. Nevertheless, it is worth emphasizing that the percentage (average 28%) of monkeys affected after consuming SEls was lower than the percentage (average 79%) affected after consuming SEA or SEB.

Although the emetic activity of SEG, SEI, SEM, SEN, and SEO in humans is not well understood. Baumgartner et al. (2014) [13] described two cases of staphylococcal food poisoning in which only isolates carrying the newly described enterotoxins genes (*seg*, *sei*, *selm*, *seln*, and *selo)* were detected. This fact indicates that a new type of SE with relatively mild vomiting activity can cause human food poisoning.

Staphylococcal enterotoxins are very stable and cannot be inactivated by methods such as heating, so it is important to prevent the growth of *S. aureus* and the production of enterotoxins in food [14]. However, it is worth emphasizing that each of the food processing elements may be a stress factor for a cell, which induces an increase in enterotoxicity. Stress conditions (a low pH value, decreased/increased temperature, or a change in osmotic pressure) result in metabolic changes in bacterial cells through the expression of specific gene sets, including enterotoxins. In this case, it is important to study the expression of each enterotoxin gene. Most studies have focused on the expression under stress conditions encountered in food processing environments of genes encoding classical enterotoxins, whereas knowledge about the expression of newer enterotoxins is limited.

Therefore, this study aimed to estimate the presence of genes coding enterotoxins in *S. aureus* by bioinformatic assay and determine the effect of the stress factors (osmotic, acidic, and alkaline stress) that occur in food and food processing environments on the expression of those genes among *S. aureus* strains. We intended to estimate whether food-related stressors have an impact on the enterotoxicity of *S. aureus*. 

## 2. Materials and Methods

### 2.1. Strains

In this study, five *S. aureus* strains isolated from cheese chain production were used. All strains belong to the collection of the Department of Industrial and Food Microbiology and have been described previously [15]. The strains were kept in Microbanks^®^ (Biomaxima, Lublin, Poland) at −80 °C until the start of analysis. Strains were resuscitated by streaking the beads on TSA (Tryptic Soy Agar; Merck, Darmstadt, Germany) and incubated statically at 37 °C for 24 h (late stationary phase) for further investigation. 

### 2.2. Gene Prediction and ANI Analysis 

Strains were chosen for whole genome sequencing and further analysis (Illumina HiSeq paired end). De novo assemblies of the *S. aureus* genomes were generated using MiSeq Illumina. The quality and completeness of the genome assemblies were assessed by testing for contamination with ContEst16S [16]. The genetic biodiversity among tested *S. aureus* strains was determined using the ANI calculator [17], which estimates the average nucleotide identity (ANI). The ANI calculator is a tool based on the alignment of genomic fragments. Online tools available from the homepage, http://genomicepidemiology.org (accessed on 20 May 2023), were used to characterize the strains.

### 2.3. Stress Determination 

The tested strains were subjected to conditions carried out under laboratory conditions related to the stress conditions that occur during food production and preservation. The NaCl concentration and pH values used in the study were chosen based on the literature and preliminary studies.

#### 2.3.1. Osmotic Stress Treatment 

The impact of osmotic stress was tested using the fresh culture in 5 mL of TSB (Merck, Darmstadt, Germany) adjusted to 4.5% NaCl. These conditions were chosen to mimic the NaCl concentration that occurs in the manufacture or preservation of food [18]. This stress was applied for 48 h (statically cultured) at 37 °C. Simultaneously, incubation in 5 mL of TSB was used as a control. The procedure was performed in duplicate. 

#### 2.3.2. Acidic and Alkaline Stress Treatment 

The effect of different pH values (pH = 4.5 and pH = 9.6) was analyzed using the culture in 5 mL of TSB with 20% (*v*/*v*) NaOH and 2 M H_3_PO_4_. TSB was mixed with NaOH or H_3_PO_4_ (sterilization was performed by filtering the additives on syringe filters (MF-Millipore™ Membrane Filter, 0.22 µm pore size) and adding them to a sterile medium under a laminar chamber) to obtain the appropriate pH value. The pH of the medium after the addition of NaOH or H_3_PO_4_ was confirmed with a pH meter (CP-501, Elmetron, Zabrze, Poland). *S. aureus* strains were grown in TSB broth as a control. This experiment was performed in duplicate to obtain two independent samples of each staphylococci strain grown under control conditions (TSB), and stress conditions (TSB adjusted pH = 4.5 and pH = 9.6). Strains were statically cultured at 37 °C for 24 h (late stationary phase). 

### 2.4. Survival Analysis 

**Figure 1:**
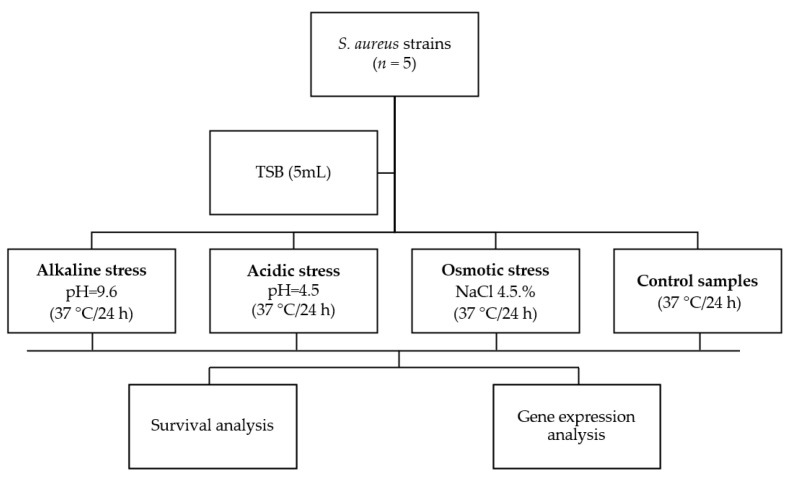
Flow chart describing the experiment’s design.

After stress treatments, the samples were divided into three equal volumes. Each sample constituted the material for conducting flow cytometry analysis, plate count method analysis, and RNA extraction (Figure 1).

#### 2.4.1. Flow Cytometer Adjustment 

A flow cytometer was adjusted as described in previous work [19]. Shortly, fluorochrome stock solutions were prepared in proportions of 1:1 of SYTO^®^9 (5 mM) and PI (20 mM). Staining of live (24 h culture) and dead (killed with 70% isopropanol) cells was performed by adding 2 mL of culture to 6 µL of dye working solutions. Then, the solutions were kept for 15 min at room temperature (20 ± 2 °C) and analyzed with a flow cytometer (FACSlyric, Beckton Dickinson, Franklin Lakes, NJ, USA). The flow cytometer’s excitation laser had a wavelength of 488 nm, and the fluorescence emissions were collected in the red and green channels. Two solutions (SYTO^®^9-stained LCS and PI-stained DCS) were used for the identification of live and dead bacteria cells and for the most appropriate cytometric parameters to avoid interference from emission spectra. Logarithmic signal amplification was employed in combination with forward scatter, side scatter, and fluorescence.

#### 2.4.2. Plate Count Method 

To check the survival rate of staphylococci strains by the plate count method, a series of ten-fold dilutions was performed for each strain before and after exposure to the tested stressors. After incubation on TSA agar at 37 °C for 48 h, the number of colony forming units (CFU/mL) was counted. 

### 2.5. RNA Extraction and Reverse Transcription 

The Total RNA Mini Plus Kit (A&A Biotechnology, Gdańsk, Poland) was used to extract a total RNA from fresh 24 h culture (as a control) and from strains subjected to osmotic, alkaline, and acidic stress. In the next step, the Clean-Up RNA Concentrator Kit (A&A Biotechnology, Gdańsk, Poland) was used for purification and concentration of the obtained RNA. To check the RNA integrity, 10 μL of RNA was loaded into a 1.2% agarose gel in 0.5% Tris/Borate/EDTA (TBE) buffer and run at 90 V for 1 h. The RNA integrity was determined by visualizing the two bands (16S and 23S RNA). RNA concentration was measured using a DeNovix DS-11 spectrophotometer (DeNovix Inc., Wilmington, NC, USA). 

After that, all RNA samples were used for first-strand cDNA synthesis using the TranScriba Kit (A&A Biotechnology, Gdańsk, Poland), using a dN-heksamer as primers. The obtained cDNA was then stored at –20 °C for further analysis.

### 2.6. Real-Time PCR Analysis of Gene Expression 

Gene expression analysis was carried out using a RotorGene Q-System (Qiagen Inc., Montreal, ON, Canada). Three independent reactions were performed for each tested gene. As the housekeeping gene, the 16S rRNA gene was chosen. For each primer pair, amplification efficiency was calculated as E = 10 (−1/slope) [20]. 

Gene expression among tested samples was analyzed using relative quantification, in which relevant gene expression is normalized to housekeeping genes, using the mathematical model proposed and validated by Pfaffi (2001) [20]. A threshold was defined using the software for real-time PCR reactions. The obtained results were interpreted as follows: values ≥ 2 indicate a significant increase in gene expression, while values ≤ 0.5 indicate a significant decrease in gene expression. 

Primers sequences for housekeeping genes (16S RNA) and enterotoxin genes according to the data from the literature, are presented in Table 1. Gene expression analysis was performed three times. The qPCR reactions were performed in a volume of 10 μL, which contained 5 μL of PowerUp SYBR Green MasterMix (ThermoFischer Scientific, Waltham, MA, USA), 1 μL of each primer (800 nM), and 10 ng of template cDNA, filled up with ddH_2_O to a final volume. The relative gene expression (REG) detected for each gene and stress condition were considered significant (*p* < 0.05) based on a t-test utilizing the RGE of the control as the reference. The real-time cycling conditions were as follows: 50 °C for 2 min, initial denaturation at 95 °C for 2 min, followed by 40 cycles of denaturation at 95 °C for 15 s and 60 s at temperature adjusted for primer set. The specificity of the real-time PCR product was determined by the construction of the melting curve by heating on a slow ramp between 60 and 95 °C in increments of 0.5 °C for 5 s.

### 2.7. Statistical Analysis 

Statistical analysis of the data obtained was carried out using the Student’s t-test and/or the one-way ANOVA at a significance level of α = 0.05 using Prism 9.4.1 (Prism, GraphPad Software, San Diego, CA, USA).

## 3. Results

### 3.1. Characterization of S. aureus Strains

All tested isolates harbored at least one SEs gene; however, only one strain possessed a *sea* gene. Other classical enterotoxins genes (*seb*, *sec*, *sed*, and *see*) were not detected. As a result of the conducted research, it was shown that the most dominant SE genes were *seg*, *sei*, *selm*, *seln*, *selo*, and *selu*. The *selp* gene was detected in two isolates (Table 2). The genetic divergence of tested *S. aureus* strains was confirmed by ANI analysis (Supplementary Table S1).

### 3.2. Survival Analysis

Calculated *S. aureus* survival after exposure to tested stress factors is shown in Table 3. The highest survival rates (mean values) of *S. aureus* were observed after exposure to pH = 9.6 (average 95.68%), while the lowest survival rate was observed in response to pH = 4.5 (average 73.04%). Thus, the greatest reduction in bacterial cells was observed after treatment with pH = 4.5 (average 26.96%). Based on statistical analysis, there were no statistically significant differences between the survival of strains obtained by plate count and flow cytometry methods (*p* > 0.05). 

### 3.3. Expression of Enterotoxins Gene 

**Figure 2:**
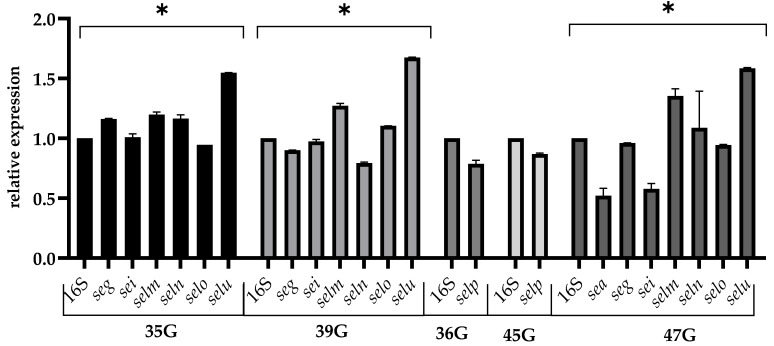
Relative expression of the enterotoxins gene among *S. aureus* strains cultured in TSB adjusted to pH = 4.5 for 24 h (late stationary phase). The data represent the mean and standard deviation of 3 independent experiments. Error bars represent SD. Asterisks denote a statistically significant difference (*p* < 0.05).

In this study, the expression of enterotoxigenic genes among *S. aureus* strains was evaluated. The most tested enterotoxins gene, after exposure to pH = 4.5 stress, has similar expression as in the optimal condition. The highest expression was observed for the *selu* gene (average 1.6). For the *selp* gene, which occurred in two strains, no significant decrease in expression levels was observed (relative gene expression levels <0.5). In the 47 G strain, under expression was noted, the value of which was 0.52 (Figure 2).

**Figure 3:**
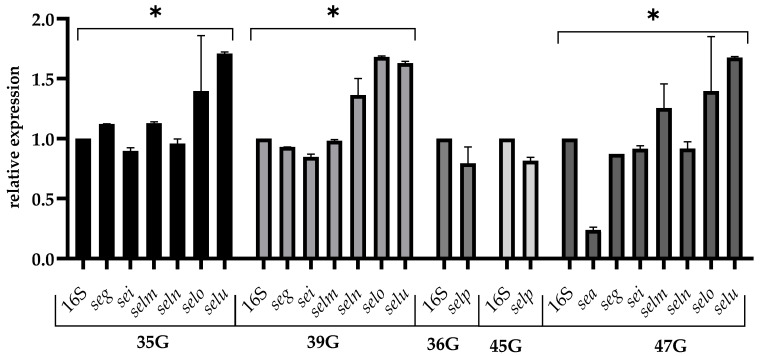
Relative expression of the enterotoxin gene among *S. aureus* strains in TSB adjusted to pH = 9.6 for 24 h (late stationary phase). The data represent the mean and standard deviation of 3 independent experiments. Error bars represent SD. Asterisks denote a statistically significant difference (*p* < 0.05).

The relative expression of enterotoxin genes after alkaline treatment (pH = 9.6) among tested strains is shown in Figure 3. Similar to acid stress, among the most tested enterotoxins genes, a similar expression as for the optimal condition was observed. A higher expression level was observed for the *selu* and *selo* genes, which averaged 1.67 and 1.32, respectively. A higher, significant decrease in expression level was observed for the *sea* gene, which was 0.21. The statistical results showed significant differences in gene expression within a strain (*p* < 0.05).

**Figure 4:**
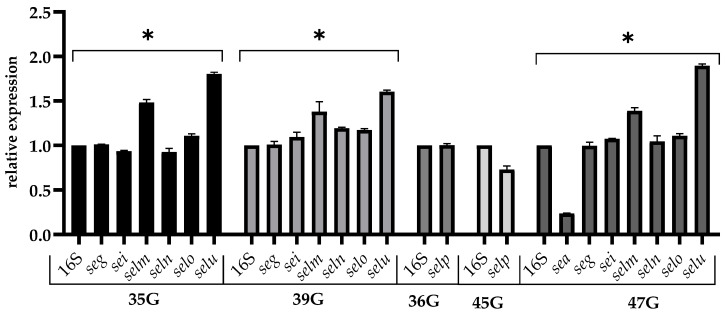
Relative expression of the enterotoxins gene among *S. aureus* strains cultured in TSB adjusted to 4.5% NaCl for 24 h (late stationary phase). The data represent the mean and standard deviation of 3 independent experiments. Error bars represent SD. Asterisks denote a statistically significant difference (*p* < 0.05).

The analysis of gene expression in response to stress caused by NaCl showed that the expression of *selp* decreased, but this decrease was not significant (relative gene expression level >0.5). In response to this stress, for *selu*, *selm*, and *selo* genes, an increase in relative expression was observed, but it was not defined as significant (average 1.77; 1.42; 1.30, respectively). Significantly decreased expression of the *sea* gene was observed (<0.5) (Figure 4).

Based on statistical analysis, statistically significant differences were observed for seg, sei, *selm*, *seln*, and *selo* gene expression after exposure to the tested stressors (*p* < 0.05; Table S3). 

## 4. Discussion

Staphylococcal enterotoxins (SEs), produced by *S. aureus*, are often responsible for staphylococcal food poisoning (SFP) worldwide [25]. In the food industry, food safety is ensured through precautions tailored to the risks associated with a particular product. These measures are based on Good Hygiene Practices (GHP) principles and Hazard Analysis and Critical Control Points (HACCP) systems [26]. 

The mentioned criteria define the maximum permitted levels of live cells of coagulase-positive staphylococci in food, and they apply during or at the end of the production process, depending on the food category. For example, the acceptable level of live *S. aureus* cells in RTE (ready-to-eat) foods should be below 10^3^ CFU/g. These rules also define that SEs must not be found in 25 g of food in any of the sample units. To guarantee the safety of food, it is crucial to establish microbiological standards for the enumeration of *S. aureus* and the detection of enterotoxins in food products. Despite their widespread use, these standards have several limitations. As one of them, the number of *S. aureus* cells is not always a sufficient indicator of the presence of enterotoxins in food. This is related to the fact that not all *S. aureus* strains are enterotoxigenic and/or express enterotoxins. It is worth noting that despite the fact that most *S. aureus* cells have been reduced using, for example, heat treatment, staphylococcal enterotoxins show resistance to high temperatures and can still show biological activity and be a causative agent of food poisoning. As per the rules, the examination of an enterotoxin is mandatory only if the values of *S. aureus* cells exceed 10^5^ CFU/g. However, recent research indicates that even in cases where this number is lower, the presence of enterotoxins can still be identified [26,27]. 

The growth and survival of *S. aureus* are dependent on a number of environmental factors. The study showed that tested strains had high survival abilities against the stress factors (osmotic, alkaline, and acidic) used in this study (73.04–95.68%). The high survival rate under various environmental conditions confirmed that *S. aureus* can easily contaminate food and potentially grow in it. Therefore, it is isolated from various food products, including milk and dairy products, meat and meat products, poultry and egg products, and seafood [28]. At the same time, it is worth mentioning that the growth dependency of SE expression is linked to regulatory elements. For example, SarA is active during the exponential growth phase, whereas Agr is active when cell densities increase during the transition to the stationary growth phase. Activation of the Agr affects leads to down-regulation of Rot, which leads to increased toxins expression [29,30].

As described previously, *S. aureus* may possess one or more enterotoxin and enterotoxin-like genes (SEs/SEls) [26]. Nevertheless, it is worth emphasizing that the harboring of enterotoxin genes does not imply that they have the capability to express them, even when provided with ideal nourishment and developmental conditions [18,31]. The reason for this phenomenon may be the complexity of the genetic expression of enterotoxin genes, which consists of a network of regulatory systems that can act both independently and in coordination [32]. Therefore, it is important to determine the impact of food-related stresses (e.g., changing pH, different temperatures, lowering the a_w_ value, as well as the addition of preservatives) on staphylococcal enterotoxicity [33]. 

One of the food preservation methods is the addition of salt. Nevertheless, *S. aureus* may survive in high NaCl concentrations, which results in the contamination of *S. aureus* in salted food that cannot be eradicated [34,35]. For all that, the concentration of NaCl might still act by affecting SE production. The presence and even growth of *S. aureus* in foods with a high content of NaCl can still be safe for consumers if enterotoxin production is sufficiently inhibited. Therefore, it is important to know the mechanisms responsible for the production of enterotoxins in the presence of NaCl [33]. 

Most pathogens, including *S. aureus,* can be inhibited when a_w_ is in the range of 0.80–0.92. For this purpose, drying or the addition of high concentrations of salt or sugar are used. The selected NaCl level used in our study mimicked the stress conditions encountered during the production of many foodstuffs (including dairy and meat products). *S. aureus* is known to be able to grow in high concentrations of NaCl (3.5 M). However, low water activity (a_w_) conditions (~0.86) have been shown to be effective in limiting SEs production [36]. Shito et al. (2015) [18] showed that 4.5% NaCl led to a decrease in *sed* expression (significant changes) among four strains, while an increase in expression of this gene was noted in only one strain. Moreover, a 30% sugar concentration caused a decreased *sed* expression, but only one strain showed significant changes [14]. In the study conducted by Etter et al. (2022) [33], equal or increased *sec* mRNA levels were found after treatment with mild NaCl stress for all tested strains. The authors showed that even a small increase in the range of 1–4.5% NaCl had an effect on *sec* expression [33]. In this study, an increase in relative expression was observed for *selu*, *selm*, and *selo* genes under 4.5% NaCl. Therefore, according to the obtained data and previously observed observations, it can be assumed that changing levels of NaCl concentration affect the transcriptional regulation of enterotoxin genes among staphylococci.

In the food industry, acid addition is used to reduce or inhibit bacterial growth. This is achieved by adding an acidifying agent as well as by enhancing natural fermentation. However, the inhibitory effect of acids depends on a number of factors, including, but not limited to, the concentration and type of acid, exposure time, and the buffering capacity of the food. Data in the literature show that, on the protein level, enterotoxin production is at its highest in a pH range between 6 and 7 [37]. Moreover, data also showed that expression of *sea* occurred under the influence of acetic acid (pH = 6.0) as a result of prophage induction [38]. On the contrary, Sihto et al. (2015) showed that lactic acid stress (pH = 6.0) affected *sed* expression, but this stress did not lead to significant changes [18]. In an older study, it was shown that mild acetic acid stress (pH 7–5.5) had an influence on prophage induction and also increased *sea* expression among tested isolates. The authors also showed that no or very low levels of *sea* mRNA and SEA were detected at pH 4.5–5.0 [38]. In the present study, it was observed that the most tested enterotoxins gene, after exposure to pH = 4.5 and pH = 9.6 stress, had similar expression as in the optimal condition.

It is worth noting that *S. aureus* is a foodborne pathogen that exhibits a competitive growth advantage in foods with high sugar or salt concentrations or a low pH. Although over the past few years many different studies [14,18,25,33,39,40] have attempted to expand on the impact of food-related stress conditions on SEs expression, there is still limited data explaining the role of stress on enterotoxins gene expression. 

Despite the fact that this study focused on the expression of genes of a newly described staphylococcal enterotoxin, based on experiments in culture media, the obtained results showed that the staphylococcal expression of enterotoxin genes may change in response to food-related stressors. Whereas the studies conducted took place under laboratory conditions using laboratory media, further studies should focus on studying the expression of SEs genes in the food matrix. Also, our study shows the expression of selected genes only in growth in the stationary phase; it is worth noting that the growth phase can also affect the expression of many virulence factors.

## 5. Conclusions

It was noted that critical food-related stressors (changing pH and NaCl concentration) had an influence on SE gene expression in *S. aureus*. This observation may be useful for risk assessment. The obtained results showed that food-related stress can influence staphylococcal enterotoxins expression, which cannot be predicted based exclusively on viable cell counts. Data about the relative expression of those genes, encoding Sels, provides information about changes in response to food and environmental stresses. It can be assumed that changes in gene expression levels under stress depend on the strain.

## Figures and Tables

**Table 1:** Primers used in this study.

Gene	Primer Sequences	Size (bp)	References
*sea*	F: TTGGAAACGGTTAAAACGAA R: GAACCTTCCCATCAAAAACA	120	[21]
*seg*	F: TTACAAAGCAAGACACTGGCTCA R: TCCAGATTCAAAYGCAGAACMAT	73	[22]
*sei*	F: GGTAYCAATGATTTGATCTCAGAAT R: GTATTGTCCTGATAAAGTGGCC	147	
*selm*	F: TCATATCGCAACCGCTGATGATG R: TCAGCWGTTACTGTCGAATTAT	150	
*seln*	F: GATGAAGAGARAGTTATAGGCGT R: ATGTTACCGGTATCTTTATTGTAT	167	
*selo*	F: GTGTAAGAAGTCAAGTGTAGAC R: CAGCAGATWTTCCATCTAACC	163	
*selu*	F: AAAATATGGAGTTGTTGGAATGAAGTT R: TTCTCTTGGGCTTTAATGTTTGTTT	201	[23]
*selp*	F: ATTTACAAAAAAAGTCTGAATTGCAGG R: TGGCGGTGTCTTTTGAACC	201	
16S RNA	F: CCGCCTGGGGAGTACG R: AAGGGTTGCGCTCGTTGC	240	[24]

**Table 2:** Characteristics of *S. aureus* strains analyzed in the study.

Isolate Code	Source	ST Type	CC	*spa* Type	Enterotoxin Genes
35 G	cheese	ST 109	CC1	t693	*seg*, *sei*, *selm*, *seln*, *selo*, *selu*
36 G	curd	ST 7	CC1	t91	*selp*
39 G	swab-form	ST 109	CC1	t693	*seg*, *sei*, *selm*, *seln*, *selo*, *selu*
45 G	curd	ST 7	-	t91	*selp*
47 G	swab	ST 5	CC5	t2	*sea*, *seg*, *sei*, *selm*, *seln*, *selo*, *selu*

**Table 3:** Results of the survival analysis among tested strains.

	pH = 4.5			pH = 9.6			NaCl		
Isolate Code	L [%]	D [%]	CFU Reduction * [%]	L [%]	D [%]	CFU Reduction * [%]	L [%]	D [%]	CFU Reduction * [%]
35 G	74.17	25.83	26.8	95.73	4.27	5.21	94.56	5.44	6.25
36 G	68.46	31.54	32.81	96.2	3.8	4.85	95.4	4.6	3.96
39 G	67.64	32.36	31.95	95.82	4.18	3.95	92.62	7.38	8.17
45 G	83.63	16.37	17.37	94.92	5.08	6.48	95.33	4.67	5.14
47 G	71.3	28.7	33.3	95.74	4.26	5.81	96.03	3.97	4.26

* plate count method L—live cells; D—dead cells.

## Data Availability

Not applicable.

## Supplementary Materials

The following supporting information can be downloaded at: https://www.mdpi.com/article/10.3390/pathogens12070954/s1, Table S1: ANI values among tested strains. Table S2; Statistical significance between expressions after exposure to tested stressor among each strains. Table S3: Statistical significance between expressions each genes after exposure to tested stressor.
