# Supplementary material for: Impact of the Food-Related Stress Conditions on the Expression of Enterotoxin Genes among Staphylococcus aureus
Source: Pathogens. 2023 Jul 19;12(7):954. doi: 10.3390/pathogens12070954 (PMC10383469; doi:10.3390/pathogens12070954)
Supplement: Supplementary file 1 [file pathogens-12-00954-s001.zip › pathogens-2474415-supplementary.pdf]

Table S1. ANI values among tested strains

	***S. aureus* 35 G**	***S. aureus* 36 G**	***S. aureus* 39 G**	***S. aureus* 45 G**	***S. aureus* 47G**
***S. aureus* 35 G**	100	98.84	99.96	98.83	98.85
***S. aureus* 36 G**	98.84	100	98.85	98.91	98.78
***S. aureus* 39 G**	99.96	98.85	100	98.87	98.96
***S. aureus* 45 G**	98.83	98.91	98.87	100	98.88
***S. aureus* 47 G**	98.85	98.78	98.96	98.88	100

Table S2. Statistical significance between expressions after exposure to tested stressor among each strains based on Kruskal-Wallis ANOVA test.

**stress factors**	**35G**	**39G**	**36G**	**45G**	**47G**
**pH=9.6**	0.00273	0.001872	na	na	0.00511
**pH=4.5**	0.002808	0.001872	na	na	0.003129
**4.5% NaCl**	0.002063	0.002041	na	na	0.00267

na- not applicable , $p<0.05$

Table S3. Statistical significance between expressions each genes after exposure to tested stressor based on Kruskal-Wallis ANOVA test.

**Gene**	***p*-value**
sea	0.06081
seg	0.019734*
sei	0.003207*
selm	0.000288*
seln	0.024283*
selo	0.000215*
selu	0.101489
selp	0.84896

*significant differences, $p<0.05$
